# The price of a vote: Diseconomy in proportional elections

**DOI:** 10.1371/journal.pone.0201654

**Published:** 2018-08-22

**Authors:** Hygor Piaget M. Melo, Saulo D. S. Reis, André A. Moreira, Hernán A. Makse, José S. Andrade

**Affiliations:** 1 Instituto Federal de Educação, Ciência e Tecnologia do Ceará, Avenida Des. Armando de Sales Louzada, Acaraú, Ceará, Brazil; 2 Departamento de Física, Universidade Federal do Ceará, 60451-970 Fortaleza, Ceará, Brazil; 3 Levich Institute and Physics Department, City College of New York, New York, NY 10031, United States of America; Central European University, HUNGARY

## Abstract

The increasing cost of electoral campaigns raises the need for effective campaign planning and a precise understanding of the return of such investment. Interestingly, despite the strong impact of elections on our daily lives, how this investment is translated into votes is still unknown. By performing data analysis and modeling, we show that top candidates spend more money *per vote* than the less successful and poorer candidates, a relation that discloses a diseconomy of scale. We demonstrate that such electoral diseconomy arises from the competition between candidates due to inefficient campaign expenditure. Our approach succeeds in two important tests. First, it reveals that the statistical pattern in the vote distribution of candidates can be explained in terms of the independently conceived, but similarly skewed distribution of money campaign. Second, using a heuristic argument, we are able to explain the observed turnout percentage for a given election of approximately 63% in average. This result is in good agreement with the average turnout rate obtained from real data. Due to its generality, we expect that our approach can be applied to a wide range of problems concerning the adoption process in marketing campaigns.

## Introduction

Elections exhibit a complex process of negotiations between politicians and voters. The past few decades bore witness to a steep increase in the expenditure of political campaigns. Take the example of the presidential elections in the US. The 1996 campaigns cost contestants approximately $123 million (corrected for inflation) altogether, an amount that escalated to nearly $2 billion in 2012 [1]. Although campaign investments have grown, the impact of money on the electoral outcome remains not fully understood [2–4], and conclusions about it are quite contradictory. In some studies, it has been argued that incumbent spending is ineffective, and the challenger spending, on the other hand, produces large gains [5–7]. Other studies claim that neither incumbent nor challenger spending makes any appreciable difference [8, 9], a theory that dates back to the 1940’s [10, 11]. Yet another group argues that both challenger and incumbent spending are effective [12].

Despite the questioning about the effectiveness of political campaigns as a whole, the election campaign of President Barack Obama in 2012 spent more than 65% of its money on media, including TV and radio air time, digital and printing advertising, and others [13]. Therefore, the direct contact with voters is not only a major factor in campaign planning, but it is believed to have relevant impact in succeeding to persuade undecided voters [14].

Here we address the problem of how campaign expenditure influences election outcome. We start by an extensive analysis of data sets from the proportional elections in Brazilian states for the federal and state congresses, uncovering a ubiquitous nonlinearity on the relation between votes and campaign budget. As we will show, candidates can be gathered into different groups of spenders. One group is characterized by candidates with low budget campaign and a seemingly uncorrelated number of votes. As the money invested on campaign increases, a clear correlation between vote and money emerges. Interestingly, in this correlated regime, the top candidates are those who spend more in political campaign, but with a highly counterintuitive result: the more the candidates spend, the less vote per dollar they get.

In Economics, a similar effect in which larger companies tend to produce goods at increased per-unit costs is known as *diseconomy of scale*. Precisely, the diseconomy of scale makes reference to a financial drawback resulting from the increase of the production scale. In the cases where a diseconomy of scale is verified, above a maximum efficient company size, the average cost per unit production increases. In other words, above this maximum, the more companies invest to increase in size, the less return of such investments they get per produced unit. The origin of this type of behavior can be manifold. For instance, it has been explained in terms of a systematic increase in communication costs [15], or as a consequence of the Ringelmann psychological effect, namely, the tendency for individuals to become less efficient when working in larger groups [16]. To the best of our knowledge, this study is the first to report the presence of diseconomy of scale on elections.

In order to elucidate the mechanisms responsible for this diseconomy in elections, we develop a general model for the negotiations between candidates and voters whose solution is compared with results from the analysis of electoral data sets. An important assumption in our model is that votes are considered to be “buyable”, whether they are somehow purchased through direct contacts between candidates and voters or, indirectly, through media campaigns. In this way, since the amount of financial resources *m*_*i*_ effectively represents the main convincing strength of candidate *i*, it also provides an upper bound for the number of votes that can be received, when competition among candidates is regarded as absent. The potential ability of a candidate to acquire votes in this model can be estimated, as a first approximation, in terms of the identification of the influential spreaders [17, 18].

A crucial goal here is to show that the competition between candidates is the root cause of the diseconomy of scale observed in Brazilian elections, mainly due to the fact that, in a scenario without competition, any model prediction will have a tendency to overestimate the number of votes of top campaign spenders. Our results show that the introduction of competition among candidates in the model combined with a simple heuristic argument lead to a prediction for the turnout rate of elections that is compatible with the average value from real data. We obtain this by the assumption that campaign planners would make use of financial resources considering an equitable division of funds per vote.

A direction not explored in this work is the explanation of the relation between money and votes where campaign funding is correlated with the vote share received in the previous election. However, the non-linearity can also be seen as indication of potential causal relationship in the direction of votes as a consequence of campaign funding. Important to note, in Brazil parties receive money campaign in proportion to the current number of deputies in the chamber, but the parties are free to choose an uneven distribution of funding among the candidates.

## Results

### Empirical findings

Our data analysis is based on real data sets acquired from recent proportional elections in Brazil, publicly available [19]. These data sets are related to the elections for the national lower house and state congress in 2014. Brazilian elections represent a quite general and suitable case study to our purposes due to a number of special factors. First, Brazil is a large country, both in population and land area. It has the fifth largest population of the world spread across roughly 8.5 million km^2^ (over 3 million mi^2^). Second, in contrast with executive elections, representative elections in Brazil have a large number of candidates. Additionally, it is compulsory to vote in Brazil. Altogether, these factors lead to a huge data set from a quite diverse electorate.

**Fig 1:**
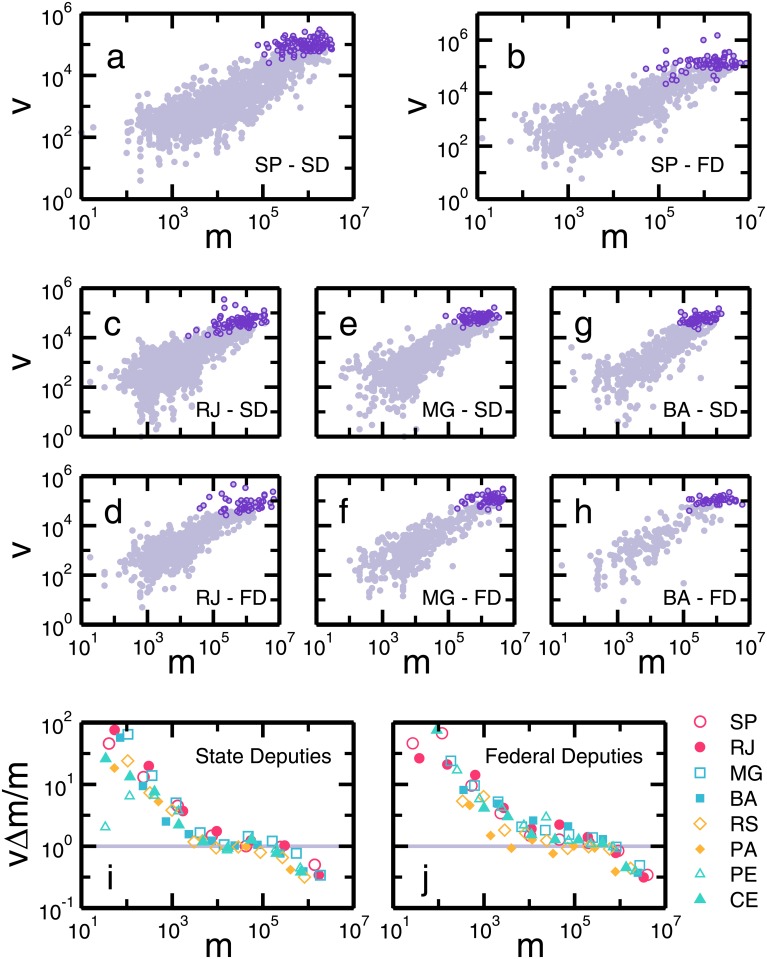
Scaling relation between number of votes and money spent. The light purple circles show the relation between the number of votes and the declared campaign expenditure of each candidate in the state (SD) and federal deputies (FD) elections in 2014 for the four largest states in Brazil: São Paulo (*A*, *B*), Rio de Janeiro (*C*, *D*), Minas Gerais (*E*, *F*), and Bahia (*G*, *H*). Despite the large fluctuations, there is an unambiguous correlation between votes and money. In each panel, the data for elected candidates are highlighted in dark purple circles. In order to see the nuances of the correlation, we plotted in a normalized relation for (*I*) state and (*J*) federal deputies for the eight largest states in Brazil. The symbols represent the normalized ratio 〈*v*〉Δ*m*/*m*, where we first calculate the average number of votes in log-spaced bins along *m*. If we assume a linear correlation, the multiplicative constant is Δ*m* = *M*/*n*. The normalization provides us a direct observation of the nonlinearity in the dependence of votes on money. We see a global sublinear behavior, where the wealthier candidates display a lower fraction of votes per money.

We start by assembling the data sets on the entire electoral outcome and campaign expenditure of candidates from all 26 Brazilian states. Fig 1 displays the number of votes *v* versus the declared campaign expenditure *m* of each candidate for the top 4 Brazilian states in terms of population, namely, São Paulo (Fig 1*A* and 1*B*), Rio de Janeiro (Fig 1*C* and 1*D*), Minas Gerais (Fig 1*E* and 1*F*) and Bahia (Fig 1*G* and 1*H*). As depicted in Fig 1, the clouds of points are neatly correlated and follow a clear trend. This trend is observed in all representative elections for all Brazilian states (see Supporting Information Section I).

To extract the main relationship between *v* and *m*, we average the number of votes in log-spaced bins along *m*, which provides an estimation for the empirical relation of *v* as a function of *m*. In order to plot results for different states in the same figure, we perform a scale transformation on *v* by supposing simple linear relation *v* = *c* × *m*, where *c* is a characteristic constant of a given election. If we define the average price of a vote as Δ*m* = ∑_*i*_ *m*_*i*_/∑_*i*_ *v*_*i*_ and suppose that it is roughly uniform across candidates, it is easy to see that *c* = 1/Δ*m*. Here, *v*_*i*_ is the number of votes of candidates *i*. If the relation between votes and money is linear, then the plot of *v* × (Δ*m*/*m*) should be a constant function of *m* with value close to 1.0.

In Fig 1*I* and 1*J*, we plot *v*Δ*m*/*m* as a function of *m* for the state legislative assembly and federal congress elections, respectively, for the year of 2014 and for the eight most populated states in Brazil. The result shows a consistent nontrivial dependence of votes on money spent in campaign. For small values of *m*, we observe a rapidly decrease of *v*Δ*m*/*m*. For intermediate expenditures in the range R$10,000 <*m*< R$100,000, we observe an apparent linear dependence of *v* with respect to *m* (at election day, October 5th, 2014, the exchange rate between the Dollar and the Brazilian Real was R$ 2.4266 = $1). Finally, for *m* > R$100,000, a noticeable departure from linearity is observed, that is, wealthier candidates need a disproportionately large amount of money to obtain a single vote as compared with less successful candidates within the same range of financial resources.

### A general model for the price of a vote

Here, we propose a general model for the price of a vote. We consider an electoral process composed of two separate groups of individuals, candidates and voters. All *s* candidates can compete for the vote of all *n* voters, and each candidate *i* has a limited amount of money *m*_*i*_ to spend on their campaigns. Thus, if at a given time *m*_*i*_ = 0, the candidate becomes unable to compete for voters anymore. Here we assume that candidates can only conquer a single vote at a given time step and that voters, once they reach a decision, cannot change their minds anymore. As compared to the case of plurality elections, the last assumption is readily justifiable for proportional elections since, in this case, candidates do not compete directly for the same seat. As a consequence, voters do not feel compelled to rethink their decisions. In this way, because it is not possible to know if a voter reached a decision or not, campaigns can spend money on already decided voters, leading to ineffective use of financial resources.

**Fig 2:**
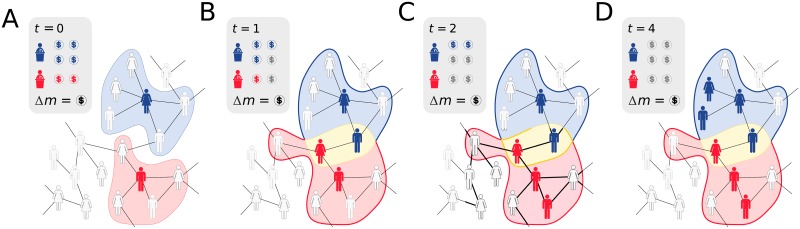
Model’s pictorial sketch. Here, two candidates compete for votes in a social network with undecided (light gray) individuals. (*A*) The blue candidate has initially a budget of 4 (four) monetary units, while the campaign for the red candidate has a budget of 2 (two) monetary units. In this example, both candidates also have one decided voter at the beginning of the process, namely, the blue voter at the top part of the network and the red one at the bottom part of the network. The highlighted regions enclose the initial voter each campaign has and the acquaintances of each initial voter. These regions represent operational areas the campaigns will act at the next time step in order to convince undecided voters to vote for their associated candidates. (*B*) One undecided voter inside each operational area is randomly chosen, becoming then decided voters. Accordingly, each budget campaign decreases by the amount of Δ*m* = $. As a consequence of new decided voters, the operational areas grow, and since two acquaintances become voters of different candidates, the operational areas of the campaigns now overlap. This region where both campaigns can act is represented in yellow. (*C*) While the red candidate’s campaign chose an undecided voter, increasing its operational area, blue campaign ineffectively spends money on a decided voter. (*D*) At the end of the campaign process, when all campaigns run out of funds, the campaign with larger initial budget ends the process with more adopters, but his/her campaign is less efficient, resulting in a diseconomy of scale.

A pictorial description of the model is presented in Fig 2. On a social network with undecided voters, represented by light gray individuals, two candidates start their campaigns with an initial amount of money *m* and one single decided voter. This initial seed is represented in Fig 2*A* by the blue and red individuals. The regions highlighted in blue and red represent the operational areas of the campaigns, enclosing the group of voters to whom the campaigns will spend money in order to turn undecided voters into decided voters. As depicted in Fig 2*B*, at each time step each campaign chooses one voter inside its operational areas. If the chosen individual is an undecided voter, she/he becomes a decided voter. Accordingly, the overall campaign money is decreased by an amount of Δ*m*. If the chosen voter is already a decided voter, as depicted in Fig 2*C*, the campaign budget is also decreased by Δ*m*, but the voter’s decision remains unchanged. We repeat this procedure until all campaigns run out of funds. In Fig 2*D*, we show a typical example of a competition for votes between two candidates during the electoral process described by our model. Although the candidate with the larger initial budget receives more votes at the end of the election, due to ineffective spending, the campaign of the poorer candidate is, in fact, more efficient.

In order to represent the reach of the traditional and social medias, as a first approximation, we apply this model on a complete graph, so that the time evolution of the number of votes of a given candidate *i* can be written as

$$\begin{array}{c} \frac{dv_{i}}{dt}=(1-\frac{S(t)}{n})\left[m_{i}\left(t\right)>0\right], \end{array}$$ (1)

where $S\left(t\right)=\sum_{i=0}^{s}v_{i}$ is the total number of decided voters at time *t*, and [*m*_*i*_(*t*) > 0] is the Iverson bracket, which is 1 if the condition inside the brackets is satisfied, and 0 otherwise. The right-hand side of the Eq 1 is the probability of candidate *i* to choose an undecided voter at time *t*. Eq 1 explicitly requires a definition for the rate of money expenditure, *dm*_*i*_/*dt*, which determines the gradual decrease in financial resources of candidate *i*. As simplifying assumptions, we consider that the amount of money spent during the campaign decreases linearly, *dm*_*i*_/*dt* = −Δ*m*_*i*_, and that this constant rate is the same for all candidates, Δ*m*_*i*_ = Δ*m*, ∀*i*.

The probabilistic feature of Eq 1 is central to confirm our hypothesis that electoral outcome is an output of campaign expenditure due to a competition process. This is shown here by first considering the case without competition, where *S* ≪ *n*. Also, we assume that *n*Δ*m* ≫ *m*_*i*_ for all *i*, so that the candidate with the highest amount of funds does not have enough money to reach out the whole network. By doing so, it is unlikely that the extent of the candidates’ campaigns overlap, and therefore, a candidate would not waste her/his campaign money on a decided voter of another candidate. As a consequence, since the probability of candidate *i* to conquer an undecided voter is not affected by another campaign, *S*(*t*) can be replaced by *v*_*i*_ in Eq 1, leading to an uncoupled system of differential equations, whose solution is given by,

$$\begin{array}{c} v_{i}=n-\left(n-v_{0,i}\right)e^{-m_{i}/n\Delta m}, \end{array}$$ (2)

where *v*_0,*i*_ is the initial number of votes of candidate *i*. Since *n*Δ*m* ≫ *m*_*i*_, and assuming that (*n* − *v*_0,*i*_) ≈ *n*, by expanding the exponential and taking its first order approximation, we can write the number of votes as *v*_*i*_ ≈ *v*_0,*i*_ + *m*_*i*_/Δ*m*. As we discuss next, this simple model does not suffice to explain the whole complexity of the relation between *v* and *m*. The first two regimes presented in Fig 1 can be understood in terms of this approximation. For the regime of low *m*, where the experimental data do not exhibit a clear correlation, the candidates start the race with *v*_0,*i*_ votes. Since they cannot afford a long run and/or a large expenditure, their final performance fluctuates around the initial value *v*_0,*i*_, which depends on different factors, such as free volunteer engagement. As campaign money increases, the linear part overcomes the initial number *v*_0,*i*_, and a linear regime emerges. However, in the scenario without competition, the linear behavior remains at large *m*.

We now consider the competition between candidates as a possible cause for the transition from linear to sublinear regime. Disregarding all previous simplifying assumptions and integrating Eq 1, we find

$$\begin{array}{c} v_{i}=v_{0,i}+\frac{m_{i}}{\Delta m}-\frac{1}{n\Delta m}\int_{0}^{m_{i}}S\left(m^{\prime }\right)dm^{\prime }, \end{array}$$ (3)

where the integration of the Iverson bracket over time gives the total time candidate *i* has to perform her/his campaign, *m*_*i*_/Δ*m*, and we used *dm*′/*dt*′ = −Δ*m* to change the variable of integration on the last term.

It is possible to find a differential equation for *S*(*m*′) by taking Eq 1 and summing over *i*. After solving it for *S*(*m*′) and integrating the last term of Eq 3 (see Supporting Information Section II for details of the analytical solution), we find a set of nonlinear coupled equations that must be solved, candidate by candidate, following an increasing order of *m*_*i*_ values. As a consequence, the number of votes of candidate *i* depends on the whole distribution *P*(*m*) through the integral term in Eq 3.

Eq 3 has a simple interpretation. As in the case without competition, all candidates begin their run with an initial number of votes, and those with sufficient money to keep running enter in a linear regime controlled by the rate Δ*m*/*m*. Nonetheless, as we will see next, candidates with sufficient campaign funds may start to waste their money on decided voters, a behavior that is substantiated by the presence of *S*(*m*′) in the last term of Eq 3, which encloses the competition dynamics. We consider this collective influence of the total financial resources from all candidates during the campaign as an important result, since it provides a bridge between campaign expenditure and electoral outcome, which is the basis of the remaining results that follow.

In order to obtain a solution for the model, we use as inputs the money *m*_*i*_ of each candidate *i*, obtained from data, the total number of voters *n*, an initial number of votes *v*_0_, and an estimated value for Δ*m*. As a simplification, we assume that all candidates starts in *average* with the same number of votes, *v*_0_ = *v*_0,i_. We define *v*_0_ as the average number of votes of candidates with less than R$1,000. This assumption is a consequence of the low correlation between votes and money when *m* < 1000. Therefore, we can better extract the value of *v*_0_ without a strong influence of *m*. Note that one can choose a homogeneous distribution for *v*_0,i_ without lack of generality. As depicted in Fig 1I and 1J, for *m* < *R*$1, 000, the quantity 〈*v*〉Δ*m*/*m* decays linearly, meaning that *v* is constant in average in this region. From this perspective, according to Eq (3), one can consider that the candidates start the competition for votes with an initial *v*_0_ in average, and receive more votes depending on their individual resources *m*_i_. The parameter Δ*m* is calculated as a function of the turnout rate *T* = *S*_*f*_/*n*, where *S*_*f*_ = lim_*t*→∞_ *S*(*t*) is the total number of votes at steady state. We can therefore write the final fraction of votes as

$$\begin{array}{c} T=1-e^{-M/(n\Delta m)}, \end{array}$$ (4)

where *M* = ∑_*i*_ *m*_*i*_ is the total amount of money in the campaign process. Therefore, we estimate Δ*m* using Eq 4 such that the total number of votes fits the turnout election data.

**Fig 3:**
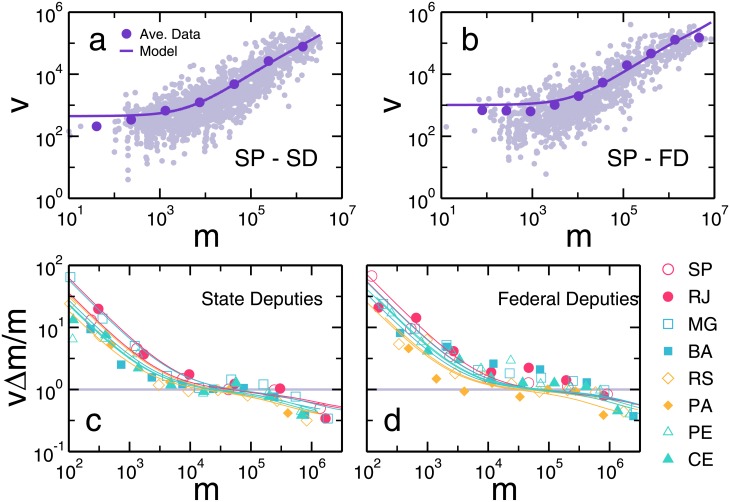
Modelling the nonlinear scaling. In order to verify if our model correctly fits the data, we show in (*A*) and (*B*) the São Paulo election for state and federal deputies in 2014, respectively. Each light purple circle corresponds to one candidate and the dark purples circles are the average number of votes in log-spaced bins along *m*. We see that our model shows a good agreement with the average behavior for all the money spectrum. In (*C*) and (*D*) we perform the same normalization process as in Fig 2 but now with Δ*m* is estimated using Eq 3. Each solid line shows the solution of our model. Despite its simplicity, our model features all nonlinear regimes seen in the data, which corroborates our theory that the inefficiency of wealthier candidates is mainly due to competition.

The results of the election in São Paulo state for state and federal deputies in 2014 are shown in Fig 3*A* and 3*B*, respectively. As depicted, the predictions of our model (solid line) are in good agreement with the average values of the number of votes for different classes of candidates in terms of fund raising. Note that we only have two parameters, namely, Δ*m* and *v*_0_. Although they have an intuitive interpretation in the context of the model, only Δ*m* has an impact for large values of *m*. For *m* < R$1,000, our model exhibits a constant behavior, capturing the uncorrelated nature of the data. Additionally, for *m* > R$1,000, an evident correlation between votes and money is present. This is better visualized when we plot in Fig 3*C* and 3*D* the normalized ratio 〈*v*〉Δ*m*/*m* for the eight most populated Brazilian states. Here, the symbols represent the data average and the lines show the solution of our model for each state identified by color. For small and large values of *m*, we see that our model exhibits a clear deviation from a linear behavior. In other words, besides exhibiting this deviation for *m* < R$1,000, a clear sublinearity is present for *m* > R$100,000. Under the perspective of our model, the observed diseconomy of scale is a direct consequence of the competition among candidates (see Supporting Information Section III for a statistical comparison between our model with competition and the linear model without competition).

Social networks are known to display the small-world phenomenon, where the typical network distance between two individuals, *ℓ*, is rather small when compared to the system size, *ℓ* ∼ log *N* [20, 21]. Our analytical solution on a complete graph works as a first approximation of such complex social network structure. In order to verify the validity of our solution, we apply the dynamics presented on Fig 2 on random graphs of different average degree 〈*k*〉 [21] (see Supporting Information Section IV). We found a good agreement between the solution on a complete graph model and the numerical simulation results obtained with a random graph model.

### Frequency distribution of votes

**Fig 4:**
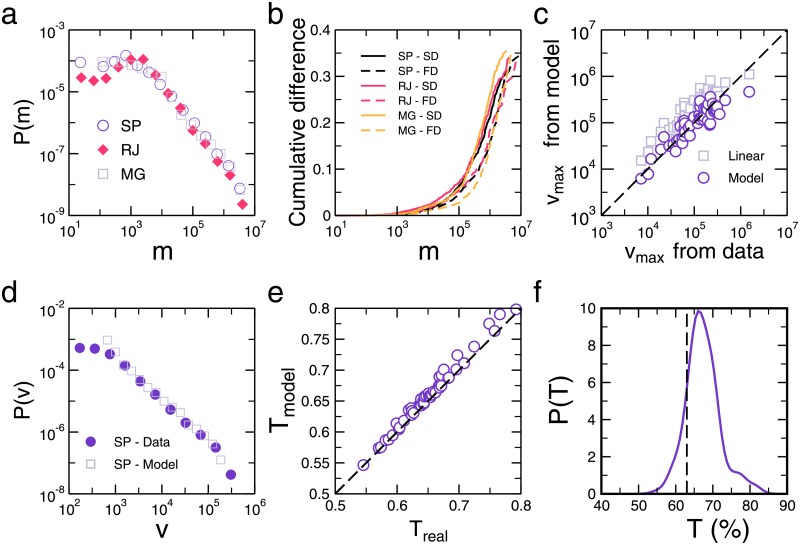
Analytical results of the model. In order to derive the distribution of votes, our model takes as input the distribution of money. (*A*) We see that the distributions of money for the state deputies in São Paulo (SP), Rio de Janeiro (RJ) and Minas Gerais (MG) reveal long tails characteristic. (*B*) We can now compare the actual distribution (circles) of votes, *P*(*v*), with the ones obtained by our model (squares) for the election of state representatives in São Paulo. Clearly, we can see that our model have a good agreement with the data showing that the universal long tail characteristic of *P*(*v*) is a direct consequence of the money as an input for dynamical competition process. (*C*) Relative difference between the cumulative distributions of the predictions for the model with and without competition. Here we show the results for the elections of state deputies (SD) and federal deputies (FD) for São Paulo (SP), Rio de Janeiro (RJ) and Minas Gerais (MG). No noticeable difference between both approaches can be observed for the region of *m* < R$10, 000. However, for the region of top spenders candidates (*m* > R$1, 000, 00), those who get elected, the relative difference is drastic, varying from 30% to 40%. (*D*) By solving our model, as expressed in Eq 1, we calculate the expected number of votes that each candidate should have for an election. The total number of votes of that election divided by the number of voters *n* is defined as the turnout ratio *T*. For all 56 parliamentary elections in 2014, we compared our estimation of the turnout ratio, *T*_model_, and the data ratio *T*_data_. The dashed line represents what would be the perfect agreement, *T*_real_ = *T*_model_. As can be seen, the simulations (circles) exhibit a good agreement with the data. (*E*) We can also pick up the candidate with the largest number of votes *v*_max_ and see how our model estimates this value. As depicted, we see that the competition model (circles) better estimates *v*_max_ when compared with the linear model (squares), which always overestimates it. The non-parametric histogram of *T* shown in (*F*) for the election of 2006, 2010, and 2014 reveals an average turnout value of approximately 67%, which is consistent with our heuristic estimation of *T* = 1 − *e*^−1^ ≈ 63% (vertical dashed line).

One of the first empirical investigations concerning Brazilian elections was carried out to determine the distribution *P*(*v*) of the number of candidates receiving *v* votes [22–25]. Since then, several other studies have been devoted to elucidate the origin of the anomalous behaviors of *P*(*v*) for other countries as well as to propose mathematical models that can provide some insight on the social and political mechanisms responsible for this statistical behavior [24, 26, 27]. In our modeling approach, however, the distribution of votes emerges as a natural outcome of the distribution of financial resources *P*(*m*). As shown in Fig 4*A*, the distribution *P*(*m*) calculated for state deputies of three different states in Brazil can all be described in terms of a power-law type of decay extending over a region of approximately three orders of magnitude. Using those distributions as inputs, we determine *P*(*v*) for each one of those elections. In Fig 4*B* we compare the empirical votes distribution for the state of São Paulo with the one obtained by our model, which reproduces correctly the empirical distribution of votes among candidates, *P*(*v*), for over two orders of magnitude (see Supporting Information Sec. V for results concerning the states of Rio de Janeiro and Minas Gerais). This implies that the observed non-Gaussian long tail form has its origin in the heterogeneous aspect of the distribution of campaign resources, regardless of the intricate social network and information dynamics behind the electoral process.

### Model validation

To highlight the effect of the sublinearity on forecasting an election, we compute the relative difference between the cumulative vote distribution predicted by the linear model without competition and the one predicted by the model with competition. As shown in Fig 4*C*, for state congress election in the top three populated Brazilian states, namely, São Paulo, Rio de Janeiro, and Minas Gerais, no significant difference is noticed between the two predictions for campaigns of low expenditure. However, for electoral campaigns that invested more than R$10, 000, a substantial discrepancy between predictions can be noticed. For this region of top spenders, the cumulative difference can be drastic, going above 30% in some cases.

We confirm the validity of our model by comparison with data from the 2014 state and federal deputy elections that took place simultaneously in the 26 states of Brazil. As shown in Fig 4*D*, where each point corresponds to an election in a given state, the model results for the turnout rate *T*, as provided by Eq 4, are compatible with the observed data. This agreement only confirms the self-consistency of our approach, since Eq 3 has been used to estimate the parameter Δ*m*. The predictive capability of the model can be effectively tested by comparing its estimate with real data for the largest number of votes obtained by a candidate in each election, *v*_*max*_. As shown in Fig 4*E*, while the results of our model (circles) gather around the identity line, demonstrating good quantitative agreement with real data, the linear approximation model, *v*_*max*_ ≈ *v*_0_+ *m*_*max*_/Δ*m* (squares), clearly overestimates the values of *v*_*max*_.

At this point, we show that our theoretical framework can provide us an estimate for the turnout ratio *T* in Brazilian proportional elections, if the following assumptions are considered: (i) the candidates have knowledge of the total amount of resources *M* during the campaign, and (*ii*) Δ*m* = *M*/*n*, which corresponds to the most simple and equitable division of votes. As matter of fact, this last point is equivalent to assume that a complete turnout can be achieved, namely, *T* = 100%, as in the case without competition. In other words, the candidates devise their strategy presupposing that they will obtain the maximum possible number of votes, therefore disregarding the competition among them. This heuristic argument leads to a fraction of valid votes, *T* = 1 − *e*^−1^ ≈ 0.63. As shown in Fig 4*F*, the histogram of the number of total valid votes for all Congress elections in the years 2006, 2010 and 2014 indicates an average turnout value of 0.67, which is in close agreement with our model prediction. Finally, we also tested our theoretical approach by applying the principle of maximum entropy [28] and found that the statistical dispersion of the model is consistent with real data from elections (see Supporting Information Sec. VI).

## Discussion

As a result of the competition between candidates in real elections, the nonlinear relation between *v* and *m* obtained here can complement other statistical analyses for political campaign and electoral outcome [29–34]. These analyses enable the detection of a number of statistical patterns of electoral processes, such as the relations between party size and temporal correlations [35], the relations between the number of candidates and voters [36], and the distribution of votes [22, 26, 27, 37]. Our approach goes beyond the examination of statistical patterns by providing a theoretical framework that clarifies a number of key issues on the economic features of electoral campaigns. First, we proposed a simple modeling framework, whose analytical solution is statistically consistent with extensive data relating financial resources of political and electoral outcomes. Interestingly, the same model also provides estimates for the distribution of votes among candidates and the electorate turnout rate that are in good agreement with real data.

A close inspection of the campaign data investigated here reveals a ubiquitous nontrivial relation between *v* and *m* for all elections investigated. More precisely, we observed that this relation is an unambiguous sublinear correlation between the money spent by candidate and her/his number of votes *v*, specially for the top spender candidates, indicating that the electoral process works in a state of diseconomy of scale. To explain this behavior in the campaign economy, we propose a general model for marketing where candidates compete with each other and must spend their money in order to get votes. Despite its simplicity, the model proves capable of reproducing the complexity of the dependence of *v* with respect of *m*. This good agreement makes our model a possible alternative to study other aspects of human collective behavior involving, for example, diffusion of innovation and decision-making, such as the competition in market share where companies invest in advertising for products.

## Supporting information

S1 File Supporting information file. In the S1 File (PDF) we present further details about the data, statistical test, and the analytical derivation.
(PDF)
