# Supplementary material for: The price of a vote: Diseconomy in proportional elections
Source: PLoS One. 2018 Aug 22;13(8):e0201654. doi: 10.1371/journal.pone.0201654 (PMC6104946; doi:10.1371/journal.pone.0201654)
Supplement: S1 File — In the S1 File (PDF) we present further details about the data, statistical test, and the analytical derivation. (PDF) [file pone.0201654.s001.pdf]

Supplementary Information: The price of a vote: diseconomy in proportional elections

Hygor Piaget M. Melo*
Instituto Federal de Educação, Ciência e Tecnologia do Ceará,
Avenida Des. Armando de Sales Louzada, Acaraú, Ceará, Brazil. and
Departamento de Física, Universidade Federal do Ceará, 60451-970 Fortaleza, Ceará, Brazil.

Saulo D. S. Reis, André A. Moreira, and José S. Andrade Jr.
Departamento de Física, Universidade Federal do Ceará, 60451-970 Fortaleza, Ceará, Brazil.

Hernán A. Makse
Departamento de Física, Universidade Federal do Ceará, 60451-970 Fortaleza, Ceará, Brazil. and
Levich Institute and Physics Department, City College of New York, New York, NY 10031, USA

* Corresponding author: hygor.piaget@ifce.edu.br

I. THE DATA

A. Data Description

In the main text we investigate the effect of the investment of candidates on campaign thanks to the available data containing the total donation received by and the expenses of each candidate. We analyze Brazilian elections for two different kinds of legislators, more specifically, the federal and state deputies. Their function is to legislate in the unicameral system of each Brazilian state. The federal deputies are representatives in the chamber of deputies of the national Congress. They are also elected for a four year term by a proportional system. The number of elected federal deputies is proportional to the population of each one of the 26 states. The data is available at the website of the Brazilian Federal Electoral Court [S1]. By force of law, each candidate must provide a detailed description of his/her campaign expenditure with specific informations such as the value, date and type of expense. All this information can be accessed by the public, however in order to know the total cost of the campaign and the number of votes of each candidate, it is necessary to process the database computationally. In Tables I and II, we show a detailed description of the data for each state. State deputies are local representatives elected for a four year term by a proportional system.

B. Results for all States

Here we summarize the results of our model for the election in 2014 of state and federal deputies in each Brazilian state. Fig S1 shows the data obtained for state deputies election and Fig S2 shows data for the federal deputies election.

II. ANALYTICAL SOLUTION

A. Calculation of the expected turnout rate T

Following from Eq (1) in the main text and summing over i, we can find a differential equation for the decided number of voters S, which reads

$$\frac{dS}{dt} = \left(1 - \frac{S(t)}{n}\right) r(t), \tag{S1}$$

where n is the total number of voters, and $r(t) = \sum_i [m_i(t) > 0]$ is the number of candidates who still have money at instant t, which depends solely on the distribution of money. After integrating Eq (S1), we find that

$$S(t) = n - (n - S(0)) \exp\left(-\frac{1}{n}\int_0^t r(t')dt'\right). \tag{S2}$$

This equation enables us to compute the expected turnout rate T of the election as a function of the average price of a vote Δm, the total money M, and n. To compute T, it is necessary to take the limit $t \to \infty$, first. At this limit, we are able to compute the value where S saturates. Then, we can define T as

$$T = \frac{1}{n} \lim_{t\to\infty} S(t). \tag{S3}$$

In order to compute the integral in Eq (S2) at this limit, we recall from the main text that $dt = -dm/\Delta m$. Then, the integral becomes

$$\lim_{t\to\infty} \int_0^t r(t')dt' = \int_0^\infty \sum_{i=0}^{N_c} [m_i(m') > 0] dm'/\Delta m, \tag{S4}$$

where N_c is the total number of candidates. After commutating the summation with the integral, and integrating the Iverson's bracket over m', we find that

$$\lim_{t\to\infty} \int_0^t r(t')dt' = \frac{M}{\Delta m}, \tag{S5}$$

which leads to

$$T = 1 - e^{-M/(n\Delta m)}. \tag{S6}$$

Fig S4A shows the turnout rate T as a function of Δm computed from Eq (S6) for the model with competition, and for the model without competition (T_{linear}). The number of votes (or money) lost by competition can be evaluated by looking at the difference between T and T_{linear}. We see that there is a maximum loss when $\Delta m = M/n$.

B. Calculation of the expected number of votes v

By integrating Eq (1) from the main text and performing a change of variables, we find that v_i can be written as a function of m_i as

$$v_i = v_i(0) + \frac{m_i}{\Delta m} - \frac{1}{\Delta m}\int_0^{m_i} \frac{S(m')}{n} dm'. \tag{S7}$$

Using Eq (S2), we can rewrite the above equation as

$$v_i = v_i(0) + \frac{1}{\Delta m}\left(1 - \frac{S(0)}{n}\right)\int_0^{m_i} \exp\left[-\frac{1}{n\Delta m}\int_0^{m'} r(m'')dm''\right] dm'. \tag{S8}$$

To find an analytical expression for v, we first decompose the external integral as

$$\begin{aligned} v_i = v_i(0) &+ \frac{1}{\Delta m}\left(1 - \frac{S(0)}{n}\right)\int_0^{m_{i-1}} \exp\left[-\frac{1}{n\Delta m}\int_0^{m'} r(m'')dm''\right] dm' \\ &+ \frac{1}{\Delta m}\left(1 - \frac{S(0)}{n}\right)\int_{m_{i-1}}^{m_i} \exp\left[-\frac{1}{n\Delta m}\int_0^{m'} r(m'')dm''\right] dm', \end{aligned} \tag{S9}$$

that compared with Eq (S8) can be rewritten as

$$\begin{aligned} v_i &= v_i(0) - v_{i-1}(0) + v_{i-1} \\ &+ \frac{1}{\Delta m}\left(1 - \frac{S(0)}{n}\right)\int_{m_{i-1}}^{m_i} \exp\left[-\frac{1}{n\Delta m}\int_0^{m'} r(m'')dm''\right] dm'. \end{aligned} \tag{S10}$$

The result of this integral relies on the limits of the external integral. Using the definition of $r(m)$ for the external interval $m' \in [m_{i-1}, m_i]$, we find that

$$\int_0^{m'} r(m'')dm'' = m_0 + m_1 + m_2 + ... + m_{i-1} + (N_c - i)m'. \tag{S11}$$

By solving the integrals, we finally find that the number of votes v_i is given by

$$\begin{aligned} v_i &= v_i(0) - v_{i-1}(0) + v_{i-1} \\ &- \frac{n - S(0)}{N_c - i} e^{-\sum_{j=0}^{i-1} m_j/(n\Delta m)} \left[e^{-\frac{(N_c-i)m_i}{n\Delta m}} - e^{-\frac{(N_c-i)m_{i-1}}{n\Delta m}}\right]. \end{aligned} \tag{S12}$$

As we can see from Eq (S12), the number of votes v_i of a candidate i is not only a function of his budget m_i, but also depends on the whole distribution $P(m)$. In Fig S4B we show how $v(m)$ changes with Δm. As Δm decreases, a large fraction of the voters become decided (i.e., $T \to 1$), and $v(m)$ displays a saturation for larges values of m resulting on the diseconomy of scale due to the competition between candidates.

III. STATISTICAL COMPARISON OF MODELS

In order to compare our model with the simple case without competition, we make use of the Akaike's Information Criterion (AIC) [S2]. The AIC is a model selection method that uses information theory to compare the relative

estimation of the information lost by mathematical models used to generate data. Here, we used AIC to measure the relative quality of our model when compared with the linear non-competitive model. Suppose that we have a model with P parameters that fits a data set with N points. Then, the AIC is defined as

$$AIC = N \ln\left(\frac{RSS}{N}\right) + 2(P+1), \tag{S13}$$

where RSS is the *residual sum of squares* given by

$$RSS = \sum_{i=1}^{N} (x_i - X_i)^2. \tag{S14}$$

Here, x_i is the ith value of the variable to be predicted and the X_i is the predicted value of x_i. We calculate the AIC for each model using Eq (S13). Then, by Akaike's criterion, the preferred model is the one with the minimum AIC value. Here, we label the model without competition as WOC and the more complex model, where there is competition, as WC. The difference in AIC is then defined as $\Delta AIC = AIC_{\text{WC}} - AIC_{\text{WOC}}$. Once this difference is computed we calculate the probability that model WC minimize the information loss:

$$P_{\text{WC}} = \frac{e^{-0.5\Delta AIC}}{1 - e^{-0.5\Delta AIC}}. \tag{S15}$$

Therefore, the probability that model WOC minimizes the information loss is $P_{\text{WOC}} = 1 - P_{\text{WC}}$. Here, we define the ratio between P_{WC} and P_{WOC} as the *evidence ratio*, which means how many times the model WC is more likely to minimize the information loss. We then performed this analysis for federal and state deputies for the 2014 elections in all 26 Brazilian states. The model WC and the model WOC are compared to the logarithm of the data (Tables III and IV), and to the data without applying the logarithm (Tables V, and VI). The AIC shows that the model with competition best explains the data when compared to the linear model in all studied cases.

IV. SIMULATION ON A COMPLEX NETWORK

In order to solve analytically the model, we make use of a mean field approximation where the network is a fully connected graph. To see if our solution still holds for a more complex topology, we performed simulations using the Erdös–Rényi network model with three different values for the average degree: $\langle k \rangle = 2$, 6 and 10. As we can see in Fig S3A and B, for federal and state deputies, respectively, we find a good agreement between the analytical solution (black line) and the real data (grey circles) for $\langle k \rangle = 6$ and 10. Due to computational performance, we chose the state of Espírito Santo to perform the simulations. First, we made use of the candidates' budget for the 2014 election as an input for the distribution of money $P(m)$. The network size is taken from the number of registered voters in Espírito Santo, $N = 2653536$, as presented in Table 1 and 2. Each candidate starts the simulation with only one node as a decided voter. This node is the initial seed for the candidate's marketing campaing. The overall underestimation of the number of votes for $\langle k \rangle = 2$ can be understood by noting that an important fraction of the network is made of unconnected nodes, therefore, for the candidates with seeds in the largest cluster the network seems to be smaller.

V. FREQUENCY DISTRIBUTION OF VOTES

Here, we show the comparison between the empirical votes distribution for the states of Rio de Janeiro (Fig S5A) and Minas Gerais (Fig S5B) with the one obtained by our model. Again, the model reproduces correctly the empirical distribution of votes among candidates, $P(v)$.

VI. STUDY OF THE DISPERSION

Our model allow us to calculate the mean or expected value of the number of votes. However, to fully describe the election we have also to study the statistical dispersion, which is given by the conditional probability distribution $p(v|m)$. We can use the concept of maximum entropy probability distribution (MaxEnt) from information theory to guess which is the $p(v|m)$ that maximizes the Shannon's Entropy [S3]. Imposing only a constraint for the mean $\langle v \rangle$, the maximum entropy continuous distribution is exponential,

$$p(v|m) = \frac{1}{\langle v \rangle} e^{-\frac{v}{\langle v \rangle}}, \tag{S16}$$

which has the property that the mean and standard deviation are the same. We see in Fig S6A that our data show a close linear relationship with approximately unit slope $\sigma \approx \langle v \rangle$, which strongly indicates that the Eq (S16) accounts for all the random variation on $v(m)$ with the expected value calculated by our model. In the inset of Fig 4F from the main text, we show these two elements in a simulation for the election of state deputy for the state of São Paulo, the greatest electoral college in Brazil. Fig S6B shows that the addition of random dispersion to our model leads to a remarkable resemblance with real election data.

[S1] http://www.tse.gov.br/

[S2] Motulskuy H, Christopoulos A (2004) Fitting models to biological data using linear and nonlinear regression: a practical guide to curve fitting (Oxford University Press)

[S3] Jaynes ET (1957) Information theory and statistical mechanics. *Phys. Rev.* 106(4):620.

	Federal deputies					
State	n	M (R$)	S_f	T (%)	r–pearson	p–value
AC	506724	8480357.97	368332	72.6888799425	0.722748043319	3.30192976139e-11
AL	1995727	18421969.9	1283120	64.2933627696	0.840392141284	4.92302048603e-31
AM	2226891	23414726.56	1560085	70.0566395032	0.905459637663	9.23656309525e-31
AP	455514	8484530.19	368061	80.8012486993	0.566853357973	1.31431785604e-10
BA	10185417	72471496.94	5982371	58.7346693807	0.698084305668	2.28399775277e-49
CE	6271554	34838910.83	4002492	63.819780552	0.737909089987	7.95861881821e-36
ES	2653536	19490814.39	1665277	62.7569024879	0.822155443552	1.7418221573e-41
GO	4331733	65145051.12	2824329	65.2009022717	0.66788295422	6.05466767683e-20
MA	4497336	21197635.67	2836788	63.0770749617	0.685587130132	8.36126780457e-35
MG	15248681	160498695.1	9273472	60.8149124505	0.806652645383	1.27631105412e-147
MS	1818937	29384486.15	1174221	64.5553419387	0.778880439738	1.8509184753e-25
MT	2189703	27179850.24	1334861	60.9608243675	0.86687644675	1.61425424251e-33
PA	5188450	19219663.68	3496764	67.3951565496	0.714596611048	4.68308734801e-30
PB	2835882	14092397.88	1773112	62.5241811895	0.855261326688	2.61029697443e-30
PE	6356307	51507676.68	4129147	64.9614154886	0.728324391535	1.46267253887e-27
PI	2345694	24898627.07	1587477	67.6762186372	0.656433873665	1.22040618126e-13
PR	7865950	69592048.16	5275880	67.0723815941	0.728660777076	6.64008626188e-52
RJ	12141145	110784215.29	7063961	58.1820001326	0.56473987424	2.1291171572e-85
RN	2327451	14178893.28	1451341	62.3575319094	0.882530044098	1.40216619215e-30
RO	1127154	16967025.91	740924	65.7340523123	0.683327785935	7.96683605323e-13
RR	299558	8358613.48	225631	75.3213067252	0.598924952323	3.49851465097e-09
RS	8392033	57254432.25	5501353	65.554472915	0.836559267138	4.74667303986e-84
SC	4859324	31716424.53	3120297	64.2125736008	0.869812045153	2.11886214421e-41
SE	1454165	8057895.72	974311	67.0014063053	0.684912931565	2.44474497848e-12
TO	996887	15619685.1	670894	67.2989014803	0.76979251044	1.60978392322e-10
SP	31998432	241919492.64	19072393	59.6041487283	0.483246969693	9.81028340891e-81

TABLE I. **Data description for Federal deputies.** Here we describe the main properties of the data for the federal deputies election from all Brazilian states. For each state we show the number of voters registered n, the total cost of the campaign in Brazilian Reais (R$) M, the number of valid votes S_f, and the turnout percentage T.

	State deputies					
State	n	M (R$)	S_f	T (%)	r–pearson	p–value
AC	506724	10656037.7	377299	74.4584823296	0.803577951964	1.59880493578e-114
AL	1995727	19627276.99	1314659	65.8736891368	0.836240190525	8.11032735735e-76
AM	2226891	28001756.68	1547128	69.4747969254	0.498718455137	4.12728400638e-39
AP	455514	5626676.58	373731	82.0459963909	0.647712202199	7.66012489633e-44
BA	10185417	47294333.36	6053428	59.432304048	0.782568469296	1.25445144149e-126
CE	6271554	32576249.09	4095292	65.2994776095	0.686934485759	7.21069456863e-83
ES	2653536	23289124.65	1748232	65.8831084259	0.741623876986	4.30172166816e-85
GO	4331733	79310623.34	2882804	66.550823885	0.734121134203	3.1534575998e-129
MA	4497336	25979148.94	2917772	64.8777854268	0.839414632196	1.67610556861e-134
MG	15248681	177676580.98	9283721	60.8821248212	0.224029765254	4.52706918339e-14
MS	1818937	45948066.57	1204007	66.1928917824	0.799004169269	6.08398880781e-90
MT	2189703	51639423.61	1375357	62.8102075944	0.771404926583	2.02454597864e-62
PA	5188450	31595425.94	3453031	66.5522651274	0.715064099361	1.78371156902e-110
PB	2835882	17219860.72	1835376	64.7197591437	0.782772014141	1.67491078919e-74
PE	6356307	40641680.29	4171737	65.6314586441	0.748176330675	1.36075786856e-90
PI	2345694	20320016.99	1607165	68.5155438007	0.816180285209	2.19894569952e-58
PR	7865950	61749634.55	5298846	67.3643488708	0.878519254427	1.18750225791e-247
RJ	12141145	130048101.34	7122375	58.6631244417	0.572037787678	5.93043422869e-167
RN	2327451	18343797.5	1529149	65.700588326	0.850127492405	3.79357570719e-72
RO	1127154	25138956.64	761590	67.5675196113	0.741913371628	3.21262552227e-70
RR	299558	13376926.76	242398	80.9185533352	0.813681589893	8.41908443078e-96
RS	8392033	54552702.15	5592657	66.6424571972	0.691245148201	3.6535374402e-98
SC	4859324	52245781.28	3280653	67.5125387811	0.816495812077	1.75144104038e-102
SE	1454165	8833829.91	967550	66.5364659444	0.716103939585	1.17133851209e-28
TO	996887	20185053.82	699008	70.1190806982	0.864802148991	1.46231152842e-78
SP	31998432	231516634.41	17618073	55.0591760246	0.722245302764	1.00059011444e-314

TABLE II. **Data description for State deputies.** Here we describe the main properties of the data for the state deputies election from all Brazilian states. For each state we show the number of voters registered n, the total cost of the campaign in Brazilian Reais (R$) M, the number of valid votes S_f, and the turnout percentage T.

		Federal deputies		
State	Δ AIC	Probability WOC	Probability WC	Evidence radio
AC	6.1827384262	0.0434646736	0.9565353264	22.0071898888
AL	4.7608349884	0.0846782011	0.9153217989	10.8094147898
AM	2.6647303199	0.2087684091	0.7912315909	3.7899967439
AP	2.806065353	0.1973353125	0.8026646875	4.0675167435
CE	10.5123485714	0.0051881611	0.9948118389	191.7465189
ES	14.4468382526	0.0007287731	0.9992712269	1371.1692559
GO	7.4677125018	0.0233425922	0.9766574078	41.8401435584
MA	7.7592626578	0.0202403068	0.9797596932	48.4063657539
MS	10.1109475602	0.0063339711	0.9936660289	156.878838813
MT	3.9037010608	0.1243517168	0.8756482832	7.0417064229
PA	9.663695483	0.0079087312	0.9920912688	125.442532017
PB	4.6657768971	0.0884355349	0.9115644651	10.3076717549
PI	2.3415470513	0.2367151943	0.7632848057	3.2244858966
RN	5.1455026334	0.0709128217	0.9290871783	13.1018221599
RO	9.807097669	0.0073655493	0.9926344507	134.767198525
RR	1.8879845033	0.2800946322	0.7199053678	2.5702219362
SC	13.3469679287	0.0012623917	0.9987376083	791.147136976
SE	2.8635043257	0.1928258238	0.8071741762	4.1860273715
TO	5.8040508651	0.0520535295	0.9479464705	18.2109931789
BA	16.0404485774	0.0003286382	0.9996713618	3041.85951117
MG	32.2756994687	9.80439653939e-08	0.999999902	10199504.8644
SP	74.5043113536	6.63123395728e-17	1	1.50801495837e+16
RJ	42.5533963117	5.74972928891e-10	0.9999999994	1739212316.23
RS	18.8727293265	7.97635097082e-05	0.9999202365	12536.0611657
PE	7.192496085	0.026694303	0.973305697	36.4611767018
PR	15.899933541	0.0003525495	0.9996474505	2835.48072726

TABLE III. **Statistical comparison between the models.** We use the Akaike's information criterion (AIC) to compare the two models: WOC (without competition) and WC (with competition). The AIC lets us determine which model is more likely to describe correctly the data and quantify by calculating the probabilities and an evidence radio. The probability column shows the likelihood of each model to be the most correctly. The evidence radio is the fraction of Probability WC by Probability WOC, which means how many times model WC is likely to be correct than model WOC. Here, the AIC was applied in the logarithm of the data.

States deputies				
State	Δ AIC	Probability WOC	Probability WC	Evidence radio
AC	40.1333350824	1.92822192099e-09	0.9999999981	518612503.667
AL	10.5644673228	0.005055382	0.994944618	196.808989398
AM	25.7812709128	2.52154694444e-06	0.9999974785	396580.948318
AP	13.4280658125	0.0012122878	0.9987877122	823.886605911
CE	24.7191563734	4.28846166679e-06	0.9999957115	233182.849525
ES	40.567665947	1.55182686878e-09	0.9999999984	644401781.26
GO	22.588786324	1.24423372754e-05	0.9999875577	80369.751722
MA	17.3075446836	0.000174437	0.999825563	5731.72803942
MS	29.9037339555	3.20986330536e-07	0.999999679	3115396.46359
MT	19.5744050013	5.61626470736e-05	0.9999438374	17804.4285563
PA	31.4296195953	1.49673445309e-07	0.9999998503	6681210.87384
PB	18.9409648861	7.7088261887e-05	0.9999229117	12971.1435601
PI	8.8288917589	0.0119565683	0.9880434317	82.6360378881
RN	8.8191591598	0.0120141936	0.9879858064	82.2348830361
RO	24.1600601847	5.67162001552e-06	0.9999943284	176315.466418
RR	20.058166039	4.4096633465e-05	0.9999559034	22676.468129
SC	30.7721830943	2.07924302228e-07	0.9999997921	4809441.61582
SE	8.7956277831	0.0121546554	0.9878453446	81.2730027198
TO	13.5362503146	0.0011485278	0.9988514722	869.679851625
BA	42.8429750252	4.97469178945e-10	0.9999999995	2010174784.34
MG	55.735515674	7.89199040127e-13	1	1.26710747119e+12
SP	168.97837878	2.0268017352e-37	1	4.93388170453e+36
RJ	82.3116713751	1.33735794708e-18	1	7.47742967531e+17
RS	52.8467317602	3.3456307332e-12	1	298897302106
PE	19.5248749346	5.75708013522e-05	0.9999424292	17368.9162859
PR	42.819329153	5.0338563126e-10	0.9999999995	1986548557.2

TABLE IV. Statistical comparison between the models. We used the Akaike's information criterion (AIC) to compare the two models: WOC (without competition) and WC (with competition). The AIC lets us determine which model is more likely to describe correctly the data and quantify by calculating the probabilities and an evidence radio. The probability column shows the likelihood of each model to be the most correctly. The evidence radio is the fraction of Probability WC by Probability WOC, which means how many times model WC is likely to be correct than model WOC. Here, the AIC was applied in the logarithm of the data.

Federal deputies				
State	Δ AIC	Probability WOC	Probability WC	Evidence radio
AC	50.1494383861	1.28880680099e-11	1	77591148589.4
AL	101.196392792	1.0604312338e-22	1	9.43012585939e+21
AM	107.771152638	3.96087877268e-24	1	2.524692265e+23
AP	120.857209596	5.70414277247e-27	1	1.75311179942e+26
CE	184.905492905	7.05151410541e-41	1	1.41813514807e+40
ES	202.215834338	1.22854055302e-44	1	8.13973944563e+43
GO	79.8068548356	4.67909285203e-18	1	2.13716639448e+17
MA	224.617775801	1.67830046988e-49	1	5.95840862792e+48
MS	118.603128883	1.76058823276e-26	1	5.67991982108e+25
MT	120.825144982	5.79633035462e-27	1	1.72522947938e+26
PA	141.83521874	1.58808440446e-31	1	6.29689453025e+30
PB	125.232526939	6.39885542365e-28	1	1.56277948757e+27
PI	105.735798465	1.09588023355e-23	1	9.12508474362e+22
RN	103.807827371	2.87353636201e-23	1	3.48003252446e+22
RO	92.3868106145	8.6787858999e-21	1	1.1522348996e+20
RR	83.7990976172	6.35707240879e-19	1	1.57305114005e+18
SC	130.020826581	5.83896996879e-29	1	1.71263083274e+28
SE	72.7348067195	1.60633972519e-16	1	6.22533318649e+15
TO	55.1303103352	1.06808352921e-12	1	936256362587
BA	342.184328822	4.96154688314e-75	1	2.01550045491e+74
MG	777.261043094	1.65923917468e-169	1	6.02685866668e+168
SP	749.737824971	1.57217132373e-163	1	6.36062994474e+162
RJ	901.70236979	1.57695115134e-196	1	6.34135051776e+195
RS	451.109474546	1.10362679252e-98	1	9.06103409936e+97
PE	142.356671451	1.22360590247e-31	1	8.1725660033e+30
PR	334.950626527	1.84669679188e-73	1	5.41507411718e+72

TABLE V. Statistical comparison between the models. We used the Akaike's information criterion (AIC) to compare the two models: WOC (without competition) and WCB (with competition). The AIC lets us determine which model is more likely to describe correctly the data and quantify by calculating the probabilities and an evidence radio. The probability column shows the likelihood of each model to be the most correctly. The evidence radio is the fraction of Probability WC by Probability WOC, which means how many times model WC is likely to be correct than model WOC.

States deputies				
State	Δ AIC	Probability A	Probability B	Evidence radio
AC	576.061906458	8.12356005108e-126	1	1.23098739187e+125
AL	238.628928458	1.52190160204e-52	1	6.57072703427e+51
AM	682.650418552	5.81226058412e-149	1	1.72050097467e+148
AP	358.738756255	1.26144655989e-78	1	7.92740677087e+77
CE	420.054263752	6.11470593755e-92	1	1.63540162064e+91
ES	480.640448515	4.26827816914e-105	1	2.34286510947e+104
GO	989.587809594	1.29938385781e-215	1	7.69595523285e+214
MA	519.730902297	1.38633608904e-113	1	7.21325808299e+112
MS	439.886900022	3.01837593293e-96	1	3.31303993347e+95
MT	310.209118004	4.35457632874e-68	1	2.29643465749e+67
PA	685.433108646	1.44574467234e-149	1	6.9168506662e+148
PB	400.461973128	1.09846804884e-87	1	9.10358750133e+86
PI	189.012621263	9.0454627114e-42	1	1.10552664016e+41
RN	249.978331952	5.2226980642e-55	1	1.91471915035e+54
RO	482.146906385	2.00969219136e-105	1	4.97588637852e+104
RR	370.038828903	4.4369982636e-81	1	2.25377595525e+80
SC	462.924309949	3.00098154818e-101	1	3.33224308096e+100
SE	139.093088068	6.2563306112e-31	1	1.59838100341e+30
TO	282.919902956	3.67048676832e-62	1	2.72443428656e+61
BA	642.920743716	2.46339667887e-140	1	4.0594355289e+139
MG	1450.44716375	1.09496501832e-315	1	inf
SP	2129.42600533	0	1	inf
RJ	1694.58149782	0	1	inf
RS	618.918508849	4.01377875167e-135	1	2.49141784306e+134
PE	369.31393498	6.37526107172e-81	1	1.56856321451e+80
PR	784.782590509	3.8603414867e-171	1	2.59044440354e+170

TABLE VI. Statistical comparison between the models. We used the Akaike's information criterion (AIC) to compare the two models: WC (without competition) and WOC (with competition). The AIC lets us determine which model is more likely to describe correctly the data and quantify by calculating the probabilities and an evidence radio. The probability column shows the likelihood of each model to be the most correctly. The evidence radio is the fraction of Probability WC by Probability WOC, which means how many times model WC is likely to be correct than model WOC.

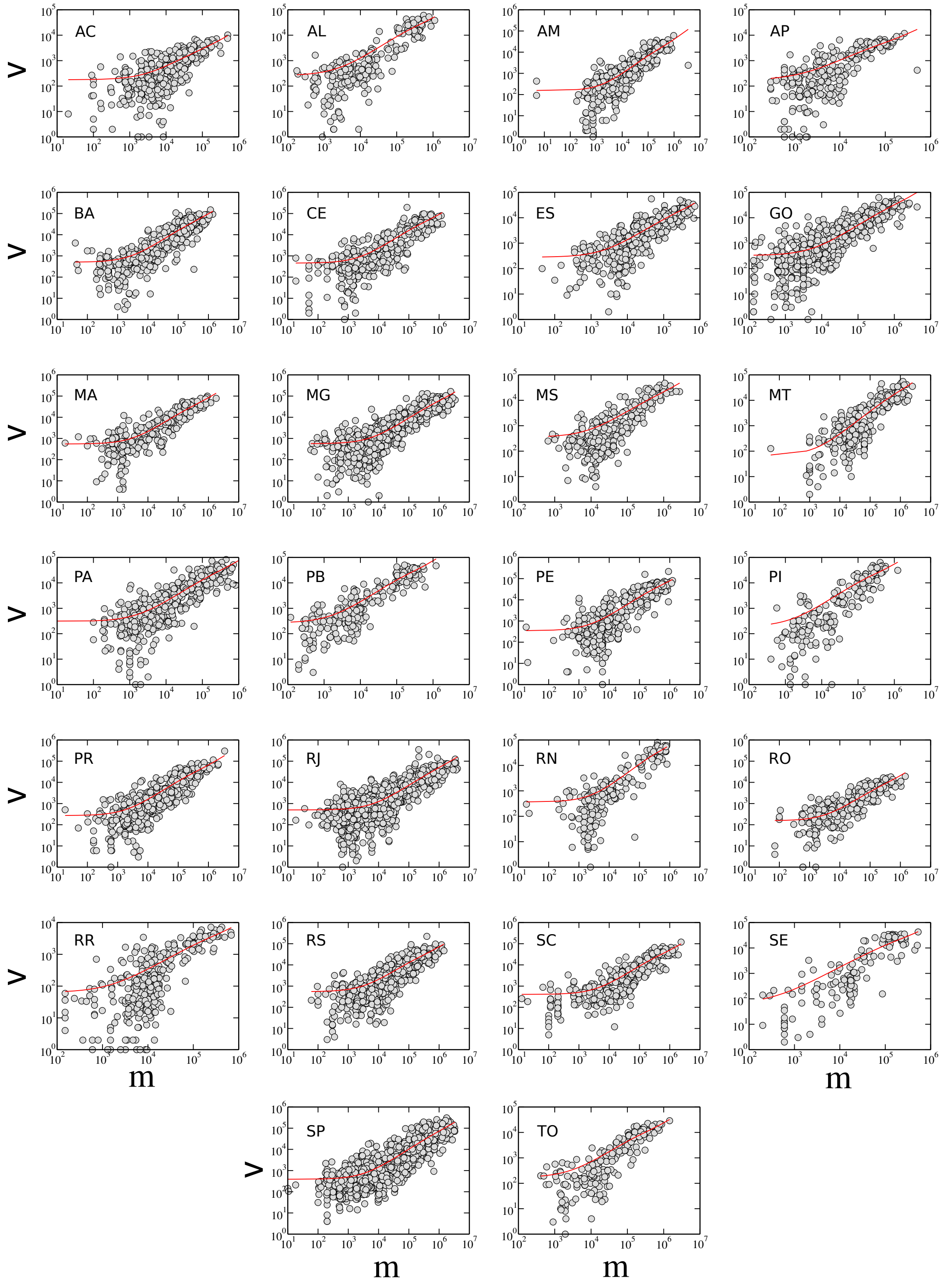

FIG. S1. **Modeling the nonlinear scaling for state deputies in all federal states.** We show how the model fits the data of state deputies election for all states in alphabetic order (AC: Acre, AL: Alagoas, AM: Amazonas, AP: Amapá, BA: Bahia, CE: Ceará, ES: Espírito Santo, GO: Goiás, MA: Maranhão, MG: Minas Gerais, MS: Mato Grosso do Sul, MT: Mato Grosso, PA: Pará, PB: Paraíba, PE: Pernambuco, PI: Piauí, PR: Paraná, RJ: Rio de Janeiro, RN: Rio Grande do Norte, RO: Rondônia, RR: Roraima:, RS: Rio Grande do Sul, SC: Santa Catarina, SE: Sergipe, SP: São Paulo, TO: Tocantis). Each gray circle represents the data for one candidate and the red line is the result of the analytical model. We see that the model shows a good agreement with the average behavior for all states.

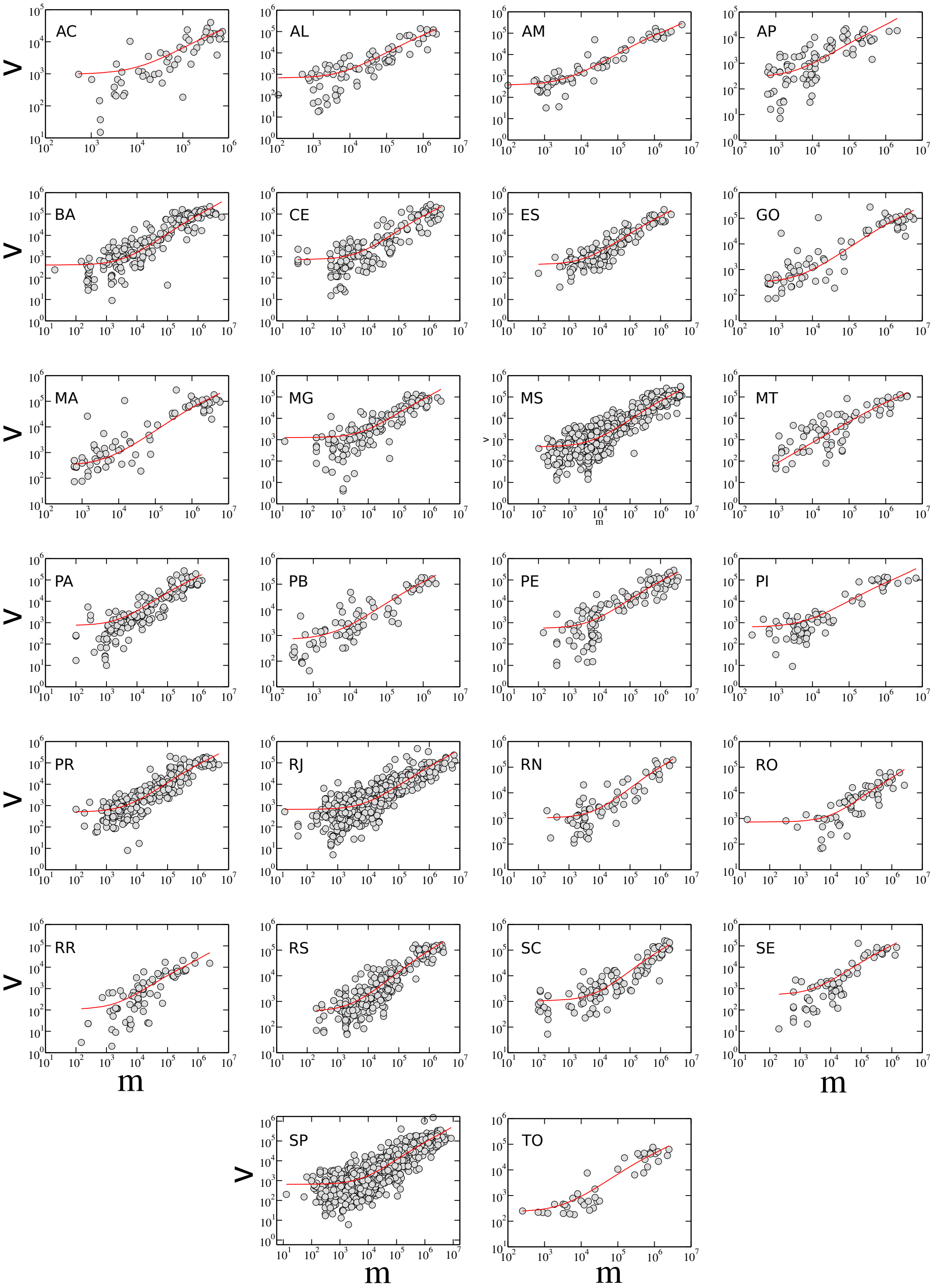

FIG. S2. **Modeling the nonlinear scaling for federal deputies in all federal states.** We show how the model fits the data of federal deputies election for all states in alphabetic order (AC: Acre, AL: Alagoas, AM: Amazonas, AP: Amapá, BA: Bahia, CE: Ceará, ES: Espírito Santo, GO: Goiás, MA: Maranhão, MG: Minas Gerais, MS: Mato Grosso do Sul, MT: Mato Grosso, PA: Pará, PB: Paraíba, PE: Pernambuco, PI: Piauí, PR: Paraná, RJ: Rio de Janeiro, RN: Rio Grande do Norte, RO: Rondônia, RR: Roraima:, RS: Rio Grande do Sul, SC: Santa Catarina, SE: Sergipe, SP: São Paulo, TO: Tocantis). Each gray circle represents the data for one candidate and the red line is the result of the analytical model. We see that the model shows a good agreement with the average behavior for all states.

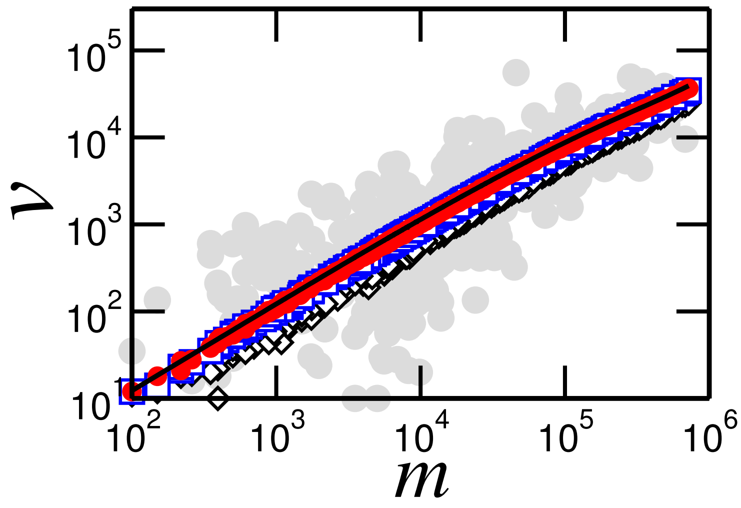

FIG. S3. **Simulation on a random network model.** Here we compare the analytical solution (black line) with the simulation on a random Erdös–Rényi network for the 2014 Espírito Santo state election of federal deputies (A) and state deputies (B). Here, each gray circle represents the data for one candidate. We used three different values of average connectivity: $\langle k \rangle = 2$ (black diamonds), $\langle k \rangle = 6$ (blue squares) and $\langle k \rangle = 10$ (red circles). Each symbol is the result of a logarithmic binning for the money (m) axis over the simulation. We see that as we increase the average network degree, the simulation presents better agreement with the analytical solution. However, the analytical solution seems to capture the overall behavior for all networks tested. The apparent disagreement for $\langle k \rangle = 2$ is a consequence of a smaller effective size of the network, since an important fraction of nodes are not connected with the largest cluster.

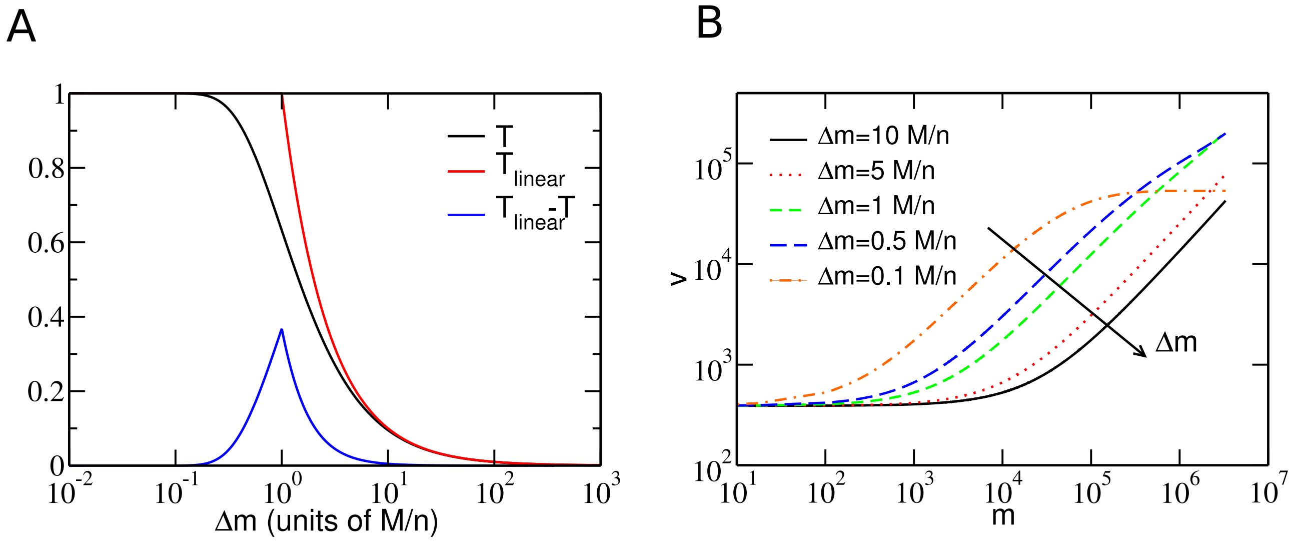

FIG. S4. **Dependence with** Δm**.** The solution of the mean field model enables us to calculate the turnout radio T as a function of the dimensionless $n\Delta m/M$ parameter. In (A) we compare the turnout for the linear case where we excluded the competition between the candidates, T_{linear}, with the case with competition, T. The competition creates an exponential saturation, which increases the waste of money when candidates seek new voters. By looking at the difference $T_{linear} - T$, we can see that this inefficiency is maximum when $n\Delta m/M = 1.0$. In (B) we show that as we decrease Δm the values of $v(m)$ usually increases, as expected by the definition of Δm. However, there is a point where a saturation appears as the total number of votes starts to get close to the size of the system, resulting on a diseconomy of scale due to the competition between candidates.

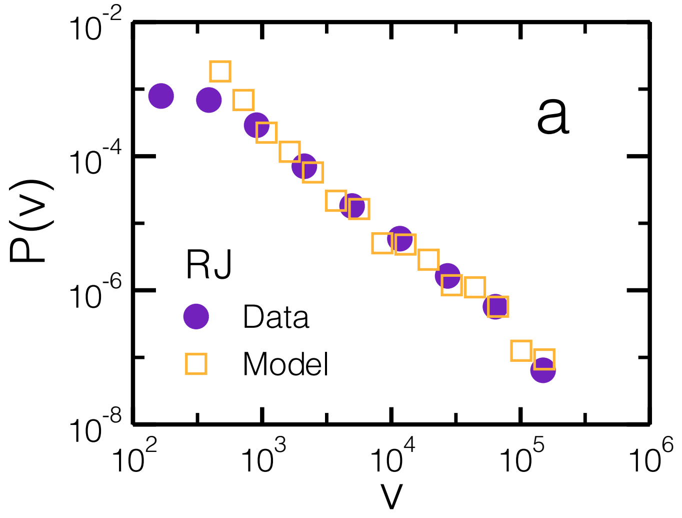

FIG. S5. **Comparison between the actual distribution of votes with the ones obtained by our model.** Here, we show the comparison for the states of **(a)** Rio de Janeiro and **(b)** Minas Gerais. Again, the good agreement indicates that the long tail of $P(v)$ is a direct consequence of the money as an input for the dynamical process.

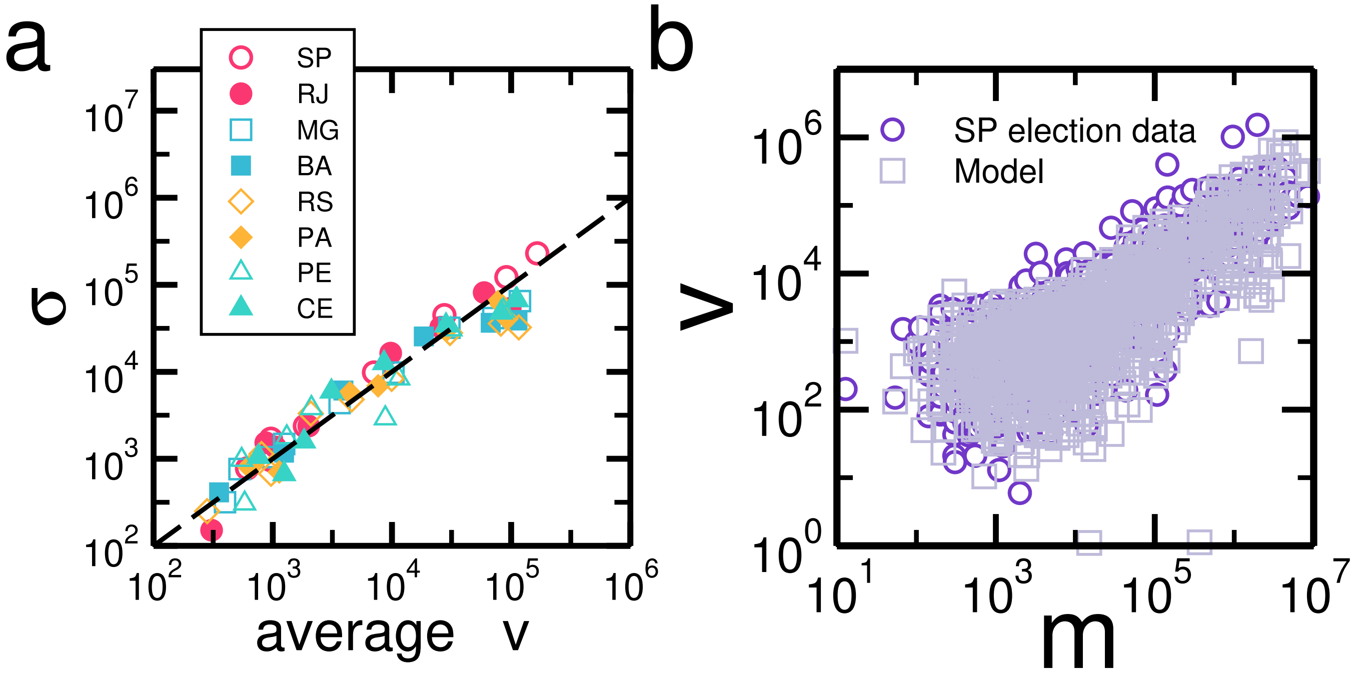

FIG. S6. **Test of statistical dispersion.** It is widely known that the exponential distribution have the property that its mean and standard deviation are equal. Therefore we use this property in order to test if the dispersion along the mean follows an exponential distribution, as predicted by the MaxEnt hypothesis. In **(a)** we see that for state deputies of the eight largest states in 2014 election the data is in close agreement with $\sigma = \langle v \rangle$ (dashed line). **(b)** of votes calculate by our model to generate a random election. Here we show for the state of São Paulo that when we add random noise to our model (squares), we obtain a cloud that closely resembles the actual data (circles).
